# Standardising the assessment of caesarean birth using an oxford caesarean prediction score for mothers with gestational diabetes

**DOI:** 10.1049/htl2.12022

**Published:** 2022-03-13

**Authors:** Huiqi Lu, Jane Hirst, Jenny Yang, Lucy Mackillop, David Clifton

**Affiliations:** ^1^ Department of Engineering Science Institute of Biomedical Engineering University of Oxford Oxford UK; ^2^ Nuffield Department of Women's and Reproductive Health University of Oxford Oxford UK; ^3^ The George Institute for Global Health Imperial College London London UK; ^4^ Women's Centre Oxford University Hospitals NHS Foundation Trust Oxford UK; ^5^ Sensyne Health Schrödinger Building Science Park Oxford UK; ^6^ Oxford‐Suzhou Centre for Advanced Research Suzhou China

## Abstract

Mothers with gestational diabetes are at increased risk of giving birth by caesarean section. A standardised assessment method may help to guide in recommendations in planning caesarean birth. We analysed 203 women with gestational diabetes managed in a single centre and developed an aggregate heuristic risk score. Among 155 women who had not had a previous caesarean birth, five risk factors (previous birth, weight gain during pregnancy, mother's height, and glycated haemoglobin and fasting blood glucose results at the beginning of pregnancy) were found associated with primary caesarean birth. Risk of primary caesarean birth in low‐risk women (score 0–1) was 13.8%, medium‐risk (score 2–3) 24.5% and high risk (score ≥ 4) 66.7%. The area under the receiver operating characteristic (AUROC) for primary caesarean birth prediction is 0.726 ± 0.003. Machine learning models were then deployed on 97 patients to explore the role of temporal blood glucose in predicting caesarean birth, achieving an AUROC of 0.857 ± 0.008. In conclusion, Oxford caesarean prediction score could help clinicians counselling women with gestational diabetes about their individual risk of primary caesarean birth. Temporal blood glucose measurements may improve the prediction subject to further validation.

## 1 INTRODUCTION

Gestational diabetes mellitus (GDM) results in hyperglycaemia (high blood glucose) of variable severity during pregnancy [1]. GDM is one of the most common medical complications during pregnancy, affecting more than ten percent of all pregnancies in the UK [2]. It is associated with a higher risk of complications during birth and a higher risk of Type 2 diabetes for both mothers and children, making GDM a condition of great public health interest in the fight against the diabetes global epidemic [3, 4].

The most common adverse effect of GDM on pregnancy is accelerated foetal growth. High blood glucose stimulates the foetal pancreas to release insulin. In the foetus, insulin acts as a growth promoting hormone. The increased perinatal risks associated with GDM include emergency caesarean birth (ECB), instrumental delivery, shoulder dystocia, and birth trauma for the baby and perineal trauma for the mother. Some of these complications could potentially be avoided through either planned elective caesarean birth (PCB), or induction of labour at an earlier gestation before the baby gets too large [5].

Unfortunately, there is no robust clinical standard to enable clinicians to confidently counsel women with GDM about their individual risk of emergency caesarean section or other complications.

Guidance from the UK's National Institute for Health and Care Excellence (NICE) recommends all mothers with diabetes in pregnancy to be counselled in the third trimester (between 27 weeks and 40 weeks of pregnancy) about the mode and time of delivery [6]. However, the guideline provided does not define in detail how to clinically decide the delivery method, or how early delivery should be performed if complications are present. As a consequence, the decision about mode of delivery is not only dependent on obstetric factors, but also greatly influenced by clinician and patient preference.

A caesarean prediction score table could be helpful to allocate healthcare resources and reduce the rate of ECB. The only model currently proposed to do this is from Thailand and has not been validated outside that setting [7]. The HAPO study [8] confirmed a linear association between blood glucose values at 28 weeks (through the oral glucose tolerance test) and subsequent risk of primary caesarean birth. Thus we hypothesise that glucose metrics during pregnancy can potentially be used to improve the prediction of primary caesarean birth.

Throughout pregnancy, women with GDM usually perform multiple capillary blood glucose tests per day to guide diabetes management. Whilst blood glucose levels are not traditionally used in clinical algorithms to recommend the mode of delivery, the advent of digital blood glucose monitoring creates opportunities for applying machine learning and other advanced signal processing methods to determine if blood glucose data can aid mode of delivery prediction.

In this paper, we address an identified clinical need for the prediction of caesarean birth, which could be used when clinicians are counselling women about their birth options (usually at 36 weeks of gestation). We demonstrate proof of concept for a new standardised assessment to predict caesarean birth in mothers with GDM. The aims of this paper are to (1) develop a caesarean prediction score for caesarean birth in women with GDM based on routinely collected electronic health records (EHR) to support birth planning, (2) use data‐driven machine learning models to evaluate whether prediction of caesarean birth can be improved by using EHR and temporal blood glucose measurements.

## 2 METHODS

In the UK, women with GDM who have had a previous caesarean birth are offered the choice of a planned caesarean birth (PCB) or spontaneous vaginal delivery (SVD). If they have developed clinical complications, such as a large baby or breech position, PCB may be recommended. However, in many countries, every woman with a previous caesarean will be offered a PCB, or alternatively, women can individually choose to opt for a PCB. Thus, following clinical recommendations, we decided first to develop a caesarean prediction score for patients who have not previously had a caesarean birth. Then, for exploratory analysis using machine learning, we have included all patients with or without previous caesarean birth to investigate the role of blood glucose in caesarean birth prediction.

### 2.1 Data preparation

Inclusion and exclusion: The proposed Oxford Caesarean Prediction Score was developed using a cohort of 203 patients who were enrolled in the TREAT‐GDm randomised controlled trial at the Oxford University Hospitals NHS Trust between September 2013 and June 2015. This was a single‐centre, individually randomised controlled trial. In this trial, temporal blood glucose measurements were collected by the home glucose management and monitoring system, GDm‐Health [9], developed by the Institute of Biomedical Engineering, University of Oxford and Oxford University Hospitals NHS Trust. The clinical trial protocol and clinical results have been published by Mackillop et al. [10, 11].

**FIGURE 1:**
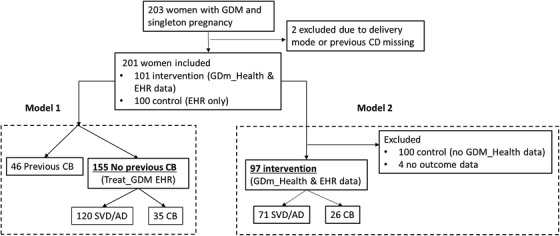
Patient inclusion and exclusion, and delivery mode review (SVD = spontaneous vaginal delivery, AD = assisted delivery, CB = caesarean birth, EHR = electronic health record)

Within this cohort, as shown in Figure 1, two patients were excluded due to missing data in either delivery mode or previous caesarean birth. There were 46 women with a previous caesarean birth and 155 women who did not. There were 102 patients recruited in the control group who did not use GDm‐Health for blood glucose recording, and 101 patients in the intervention group who used GDm‐health. 98 mothers in the intervention group completed the trial. Temporal blood glucose measurements were available for 97 patients.

### 2.2 Electronic health record and engineered features

Clinical literature suggests that the main risk factors for PCB are breech presentation and previous caesarean birth, whereas the main risk factors for ECB are foetal distress in labour, breech presentation and size mismatch between the mother's pelvis and the baby's head [3].

After considering the risk factors of ECB discussed in the introduction and the electronic health record (EHR) data collected in the TREAT‐GDm study, the following dependent factors were included in model development: age at the beginning of pregnancy, parity (number of previous births), ethnicity, glycated haemoglobin (HbA1c) prior to confirmation of GDM, weight, height, gestational days at recruitment and subsequent clinical visits, medication type, blood pressure (diastolic and systolic), oral glucose tolerance test results (OGGT, conformation test of GDM), and previous caesarean birth events.

To customise a caesarean prediction score, in model one, EHR features were changed from continuous to categorical data, and a group of engineered features were generated based on clinical literature. The threshold of blood pressure in pregnancy is based on a vital signs study at Oxford University Hospitals [12]. The hyperglycaemia (OGGT fasting, 1 h, 2 h) thresholds are based on the IADPSG criteria [13]. Weight changes are categorised based on the WHO IOM standard [14, 15]. The EHR features, engineered features, and their thresholds (for outlier filtering), are listed in Table 1.

**TABLE 1:** Category definition of EHR and engineered feature and Pearson Chi‐square test between CB and non‐CB in the primary caesarean birth cohort (*n* = 155), *p* < 0.2 for shortlisting

EHR features	Category definition	Clinical explanations	Categorical names	Number of patients	χ^2^ test (*p* value)
Parity	0 [1,10]	Nulliparity Multiparity	**ParityGainCat (0)** **ParityGainCat (1)**	76 (49.0%) 79 (51.0%)	**0.025**
Maternal height, cm	[80, 160] [161, 240]	Shorter woman has smaller pelvis, hence increase risk of CSB at labour. Threshold of 160 is based on histogram observation.	**HeightCat (0)** **HeightCat (1)**	51 (32.9) 104 (67.1)	**0.025**
Maternal weight gain, kg	WHO IOM standard [14]	Within recommendation Above recommendation	**WeightGainCat (0)** **WeightGainCat (1)**	70 (46.2%) 85 (54.8%)	**0.064**
Maternal age, years	[15,34] [35,50]	N/A N/A	AgeCat (0) AgeCat (1)	83 (53.5%) 72 (46.5%)	0.628
HbA1c, g/L	<5.6 ≥5.6	Normal High	**HbA1CCat (0)** **HbA1CCat (1)**	111 (71.6%) 44 (28.4%)	**0.010**
Systolic blood pressure, mm Hg	[60–140] [10–250]	Not hypertension hypertension	SBP_Cat (0) SBP_Cat (1)	153 (98.7%) 2 (1.3%)	0.351
Diastolic blood pressure, mm Hg	[40–90] [90–150]	Not hypertension hypertension	DBP_Cat (0) DBP_Cat (1)	153 (98.7%) 2 (1.3%)	0.442
OGGT fasting, mmol/L	<5.1 [5.1,6.9 ] ≥7	Normal Impaired Hyperglycaemia	**FastingCat (0)** **FastingCat (1)** **FastingCat (2)**	76 (49.0%) 74 (47.8%) 5 (3.2%)	**0.007**
OGGT 1 h, mmol/L	10.00	Normal Abnormal	OneHr_Cat (0) OneHr_Cat (1)	120 (77.4%) 35 (22.6%)	0.614
OGGT 2 h, mmol/L	<7.8 [7.8–11] >11	Normal Impaired Hyperglycaemia	TwoHrCat (0) TwoHrCat (1) TwoHrCat (2)	102 (65.8%) 48 (31.0%) 5 (3.2%)	0.224
Medication group one	No BG control medication BG controlling medication*	Diet and exercise Medication: either Metformin** or insulin	Med_Cat (0) Med_Cat (1)	82 (52.9%) 73 (47.1%)	0.568
Medication group two	Non‐insulin Medication with insulin	Diet, exercise or Metformin** Medication with insulin	MedInslin_Cat (0) MedInslin_Cat (1)	123 (79.4%) 32 (20.6%)	0.713

^*^The patient has medication since the confirmation of GDM or new blood‐glucose‐related medication subscription after the confirmation of GDM where previously their blood glucose state was normal.

^**^Metformin lowers blood sugar levels by improving the way body handles insulin. It is usually prescribed for diabetes when diet and exercise alone have not been enough to control blood sugar levels.

In model two, training and testing data in the model include routinely collected EHR data (instead of the complete list of EHR collected in the TreatGDM clinical trial), blood glucose measurements collected through GDm‐Health, and engineered blood glucose features. Due to the limited number of patients in our data, we engineered blood glucose features instead of using raw daily blood glucose readings. The moving average method was used to deal with missing values. During the Treat‐GDm clinical trial, patients have the option to take up to six blood glucose tests every day. These readings were tagged as before‐ or after‐ breakfast, lunch and dinner. “Mean32less” and “Mean3336” are the mean blood glucose of all six‐tag readings at or before 32 weeks of pregnancy and between 33 and 36 weeks of pregnancy.

We engineered two groups of engineered features based on the frequency and importance of the fasting (before‐breakfast) blood glucose reading. “BBWeek 1–6” are six weekly‐average fasting blood glucose values before the last blood glucose measurements. “BBW1ROI ‐ BBW6ROI” are the weekly average of out‐of‐threshold fasting blood glucose readings. ROI stands for the region of interest, in our case, the region beyond the normal blood glucose range. These ROI features were engineered to represent the cumulative effect of excessive blood glucose.

### 2.3 Caesarean prediction score development and modelling

There are two modelling stages in the development process.

Model one is an aggregate caesarean prediction score for the prediction of primary caesarean birth for mothers without previous caesarean. Training and testing data are physiological measurements already recorded in the TREAT‐GDm study and in routine practice when a patient is being monitored in hospital settings.

Firstly we carried out a histogram observation and tests of homogeneity of variances using Levene tests on all the continuous dependent factors before converting them into categorical features. Continuous factors that were shortlisted by using chi‐square tests were then changed into categorical features based on clinical definitions (if available) or observed distributions, as defined in Table 1.

Secondly, a generalised linear logistic regression model with a backward stepwise conditional method was applied. The condition used in the backward stepwise was that the *p*‐value in the Chi‐square tests between the categorised risk factors and the binary delivery outcome, namely caesarean section or vaginal birth, needed to be smaller than 0.2 to be included in the final model.

Thirdly, a caesarean prediction score table was generated based on the coefficients estimated in the logistic regression model. A score was allocated to each risk factor, with the magnitude of the score reflecting how extremely the parameter varies from the norm. The area under the curve (AUROC) was used to evaluate the performance of the prediction.

Model two focused on using data‐driven machine learning approaches to predict the delivery mode. The machine learning models tested include a generalised linear regression model (GLM), a support vector machine (SVM), a random forest (RF), and ensemble boosting models, including AdaBoost, GentleBoost, LogitBoost (the best performance of these three models was reported). The training and testing pipeline of machine learning models is shown in Table 2. For feature selection, Lasso regularisation was used in the logistic regression model, backward stepwise was used in SVM and ensemble boosting models, and Shapley values were used for RF model. Grid search, random search and Bayesian optimisation methods were used in hyperparameter optimisation within the MATLAB Machine Learning Toolbox.

**TABLE 2:** Training and testing pipeline of developing LR, SVM, RF and boosting machine learning models with downsampling and model evaluation based leave‐one‐out and AUROC

1	Pre‐processing: missing data imputation, select windows of observation
2	Feature selection and hyperparameter tuning of each ML method, using the whole feature set: opts.SelectedVariableNames = [“HbA1c”, “Age”, “Parity”, “BookingBMI”, “Previouscaesareansection”, “SBPatrecruitment”, “DBPatrecruitment”, “Highestmaternalweight”, “Fasting”, “OGGT1hour”, “OGGT2hour”, “Medicationtype”, “BBWeek1”, “BBWeek2”, “BBWeek3”, “BBWeek4”, “BBWeek5”, “BBWeek6”, “BBW1ROI”, “BBW2ROI”, “BBW3ROI”, “BBW4ROI”, “BBW5ROI”, “BBW6ROI”, “Mean32less”, “Mean3336”]
3	Feature selection, then update each ML model with selected features and hyperparameters
4	Initialise training
5	Randomly downsampling Group 0 (non‐CB patients) data into three‐fold, then use the leave‐one‐out for cross‐validation
	Training and evaluation: down_sample_ratio =3 For loop = 1:10 % ten loops to evaluate down‐sampling For loop2=1:datasize % total size of data For loop3= 1:down_sample _ratio Train models: LR,SVM, RF, boosting End End Record AUROC, sensitivity, specificity, precision, recall and F1 score End
6:	Plot AUROC for model selection and report results

Data downsampling was performed to address unbalanced data (in a ratio of 3:1 for non‐CB: CB) before model training. Model performance was assessed using the leave‐one‐out method. To account for the relatively small dataset, the leave‐one‐out method was repeated ten times to achieve a reliable measure of model performance. The area under ROC curve (AUROC), sensitivity and specificity were reported to evaluate the performance of models.

## 3 RESULTS

In model one, 155 patients were included in the development of a caesarean prediction score for women without previous caesarean birth. Among them, 120 delivered under mode one (SVD and assisted delivery) and 35 patients delivered under mode two (either planned or emergency caesarean).

As explained in Table 1, continuous variables were categorised into categorical features based on clinical standards, clinical evidence, or distributions if a clinical reference was not available. The result of the Pearson Chi‐Square Test between caesarean and vaginal birth in the Primary caesarean birth cohort (Table 1) provides the estimated probability of correlation between delivery mode and risk factors. *p* < 0.05 indicates a significant correlation, and *p* < 0.2 is used for shortlisted risk factors.

**FIGURE 2:**
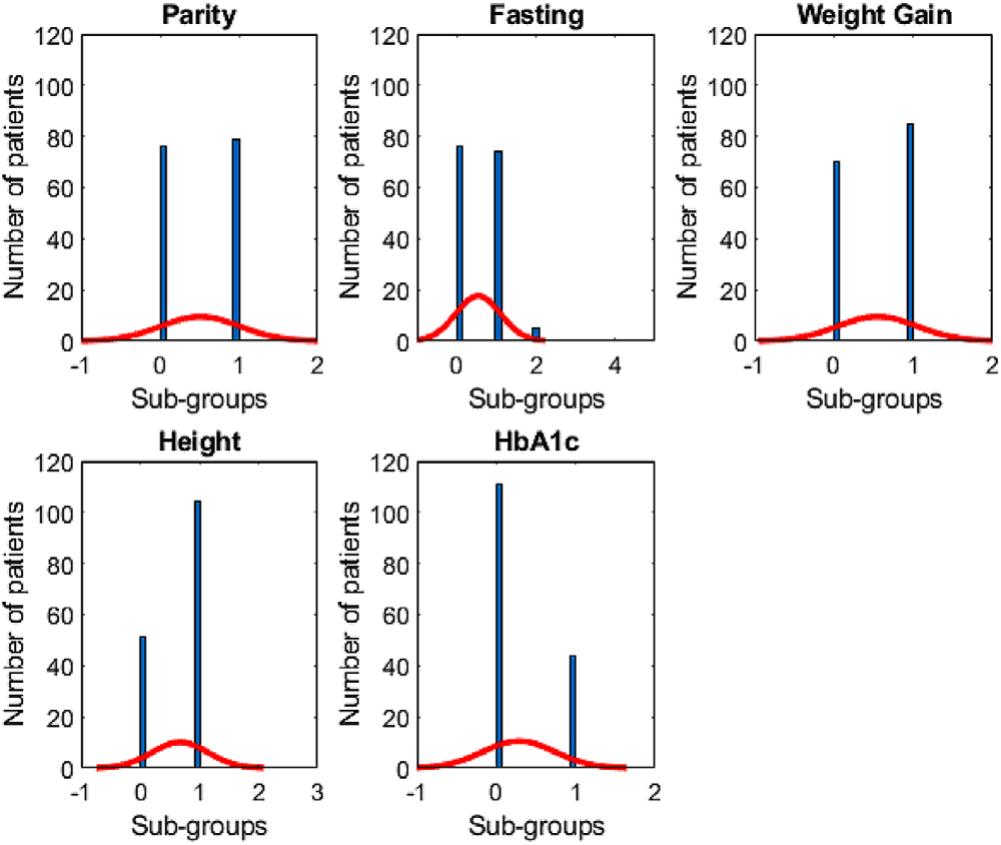
Distribution of sub‐groups that corresponding to Oxford caesarean prediction score

There were six risk factors shortlisted (highlighted in bold in Table 1). Then we trained a generalised linear logistic regression model using the backward stepwise conditional method. The five features were selected by the final model include ‘WeightGainCat’, ‘HeightCat’, ‘HbA1C’, ‘ParityGainCat’, and ‘FastingCat’. The distributions of sub‐groups of the selected features are shown in Figure 2. The caesarean prediction score table with corresponding sub‐group and scores are listed in Table 3. The caesarean prediction score of each selected predictor was calculated according to its coefficient value divided by the smallest absolute coefficient value, rounded to the nearest integer. The odds ratio is the exponential of the coefficient.

**TABLE 3:** Oxford caesarean prediction score for mothers with gestational diabetes

	Coefficient	Sig.	Odds ratio 95% C.I.	Score
WeightGainCat(1)	0.686	0.14	1.99 (0.80, 4.94)	1
WeightGainCat(0)	Reference			0
HeightCat(0)	1.106	0.01	0.33 (0.14, 0.79)	2
HeightCat(1)	Reference			0
HbA1cCat(1)	0.862	0.06	2.37 (0.98, 5.71)	1
HbA1cCat(0)	Reference			0
ParityGainCat(0)	0.619	0.15	0.54 (0.23, 1.26)	1
ParityGainCat(1)	Reference			0
FastingCat(0)	Reference			0
FastingCat(1)	−0.070	0.88	0.93 (0.37, 2.33)	0
FastingCat(2)	2.520	0.04	12.42 (1.127, 137.02)	4

**FIGURE 3:**
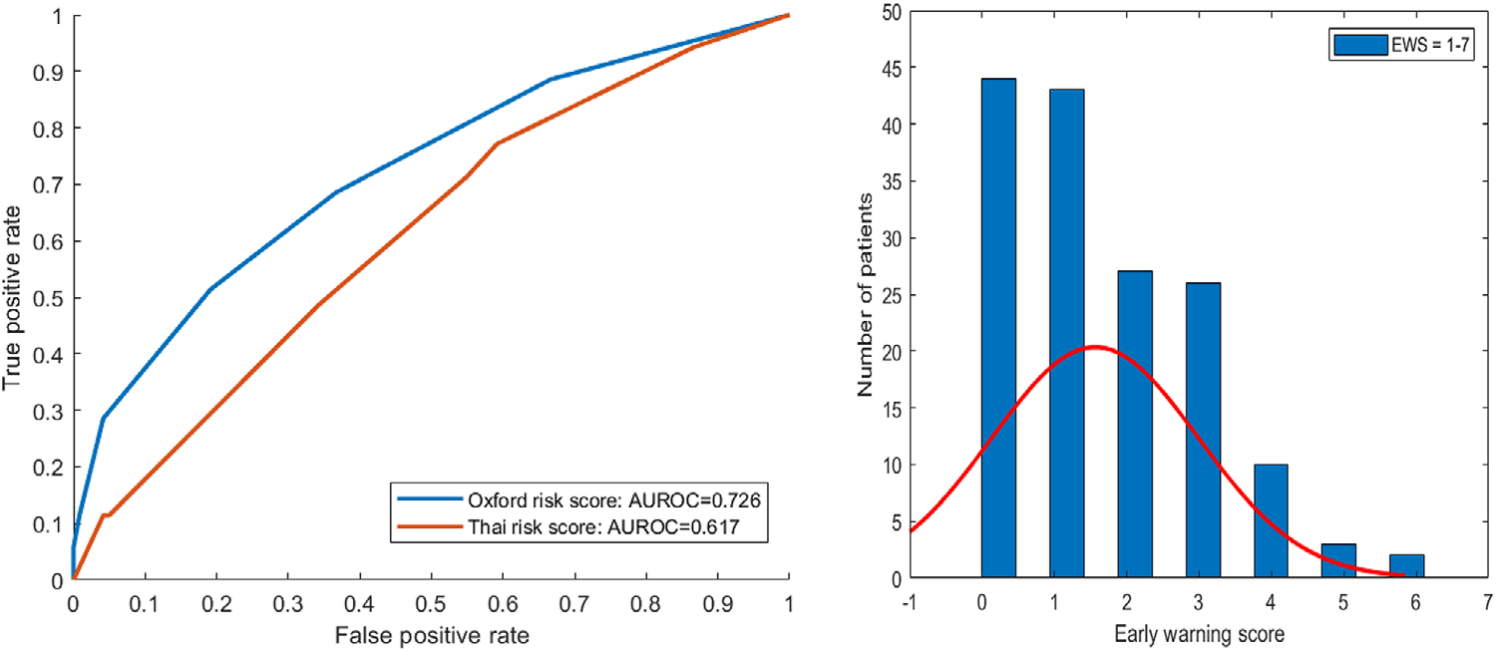
Oxford caesarean prediction score performance and distribution

The AUROC of the Oxford caesarean prediction score for primary caesarean birth is 0.726 ± 0.003. The distribution of caesarean prediction scores is shown in Figure 3. To evaluate the difference in caesarean prediction scores between different populations, we also tested a risk score for primary caesarean birth developed based on a Thai population [7]. The AUROC of the Thai model on the UK cohort is 0.617 ± 0.005.

Based on the distribution of risk scores and the clinical explanation of the combination of risk factors, we developed a three‐tier risk score system. Low risk was defined as 0–1 point, medium risk is defined as 2–3 points, and high risk is 4 points and above. The performance of applying this risk score to the UK cohort is shown in Table 4.

**TABLE 4:** Oxford caesarean prediction score risk groups and percentage of patients underwent primary caesarean birth (CB)

Risk group	Total number of mothers	Mothers undergone primacy CB
Low (0–1 point)	87	12 (13.8%)
Medium (2–3 points)	53	13 (24.5%)
High (4 points and above)	15	10 (66.7%)

In model two, 97 patients with 42,805 longitudinal blood glucose readings were recorded. After removing 1008 invalid readings (2.4%), 41,797 readings were included in the model training and evaluation. Among the valid readings, 19.7% were collected before breakfast, 16.3% after breakfast, 15.9% before lunch, 15.3% after lunch, 16.7 before dinner and 16.2% after dinner. Feature selection results, listed under groups of ‘EHR features’ and ‘BG features’ were reported in Table 5. SVM and Boosting shared the same selected features.

**TABLE 5:** Feature selections in machine learning models

Feature selection	Selected features
Generalised linear model Lasso regulation	EHR features: ‘Previouscaesareansection’, ‘OGGT2hour’, ‘HbA1c’, ‘Fasting’ BG features: ‘Mean3335’, ‘BBWeek1’
SVM Backward stepwise	EHR features: ‘Previouscaesareansection’, ‘HbA1c’, ‘Fasting’, ‘OGGT2hour’, ‘BookingBMI’, 'Medicationtype‘ BG features: 'BBW1ROI1', ‘Mean3335’
Ensemble boosting Backward stepwise	EHR features: ‘Previouscaesareansection’, ‘HbA1c’, ‘Fasting’, ‘OGGT2hour’, ‘BookingBMI’, 'Medicationtype’ BG features: 'BBW1ROI1', ‘Mean3335’
Random Forest Shapley value	EHR features: ‘Previouscaesareansection’, ‘HbA1c’, ‘BookingBMI’, 'OGGT2hour’ BG features: ‘Fasting’, 'BBWeek1', ‘BBWeek2’, ‘BBWeek3’, ‘BBWeek4’, ‘BBWeek5’, ‘BBW2ROI’

Model performance based on AUROC, sensitivity and specificity was reported in Table 6. Among these models, the GLM model using logistic regression with Lasso regulation provides the best AUROC score. The performance of the SVM and RF models were similar, while ensemble boosting resulted in the poorest performance among the ML methods tested.

**TABLE 6:** Data‐centric models, selected features and performance

Models	AUROC	Sensitivity	Specificity
Generalised linear model	0.857 ± 0.008	0.935 ± 0.021	0.630 ± 0.051
SVM	0.833 ± 0.007	0.742 ± 0.008	0.764 ± 0.031
Ensemble boosting	0.801 ± 0.019	0.739 ± 0.011	0.728 ± 0.038
Random Forest	0.825 ± 0.007	0.757 ± 0.020	0.733 ± 0.020

Results suggest that (1) there is a potential to generate a robust and accurate risk score table on delivery mode for expecting mothers with GDM, including patients with previous caesarean birth, (2) based on the ranking of feature importance in all ML models, blood glucose plays a significant role in delivery mode prediction, (3) based on the results of ML models, we need a larger dataset to allow boosting methods and tree methods to outperform GLM, as current boosting and tree models are overfitted.

## 4 CONCLUSION AND DISCUSSION

This paper provides a proof of concept that prediction of caesarean birth can be done using a caesarean prediction score for mothers with GDM, and that inclusion of engineered blood glucose features can improve model performance. We propose a pragmatic approach, with an emphasis on system‐wide standardisation and the use of physiological parameters that are routinely measured in NHS hospitals and prehospital care. We developed one clinically‐plausible standardised caesarean prediction score chart, in line with the current NICE guidelines for caesarean prediction, then used data‐driven machine learning models that incorporate temporal blood glucose data, to explore the association between diabetes and delivery mode.

One main challenge of providing an assessment of caesarean birth is understanding the reasons for large variations in caesarean rates in maternity clinics in the UK and worldwide. Among the UK NHS Trust hospitals, the rates of caesarean section for general maternity clinics are ranges from 13.6% to 31.9% [3]. There is a lack of data specifically from maternity diabetes clinics in the UK, but the range of caesarean birth rates for women with diabetes is expected to be large. A study in Denmark also suggested similar results, where the proportions of caesareans had systematic variation between hospital units. [4] This demonstrates the urgent need for methods of standardising the advice women receive and benchmarking practice. We believe a National Caesarean Prediction score for caesarean births could be a valuable tool for these purposes.

Based on discussions with clinicians, we suggest the differences in caesarean birth rate in the UK are caused by the variation of interpretation of the NICE guidelines, such as intrapartum care provision, advice given to mothers, and patient demographics. For women with an indication for caesarean birth, for example, breech, personal request, there is typically no ambiguity in clinical decision‐making for delivery mode. However, there is a lack of standard guidelines for the majority of mothers who do not have an absolute indication for caesarean birth. Clinical equipoise is most marked in women who have not had a previous caesarean section. This is why we developed the caesarean prediction score in model one, to address clinician's need for further information to inform decision making. The ultimate goal is to reduce the proportion of women undergoing ECB.

The strengths of this study are the use of NICE‐guided management of diabetes in pregnancy, and use of the international WHO/IADPSG thresholds for GDM diagnosis. Whilst this is a secondary analysis of data from a clinical trial, the data were collected under “real‐life” conditions in a busy maternity diabetes service. We considered a range of clinical and non‐clinical outcomes, important for comprehensively evaluating the potential benefits of a caesarean prediction score system. We also present the first risk score table for caesarean birth in the UK cohort. We assessed the feasibility of using a caesarean prediction score that was previously developed based on a Thailand cohort to the UK cohort. The differences in model performance, as shown in Figure 2 were most likely reflect the different demographics, risk factors and clinical practice between the UK and Thailand. The Thai risk score table included the number of births given before the current pregnancy, mother's weight gain during pregnancy and insulin use, corresponding to risk factors ‘ParityGainCat’, ‘MedInslin_Cat’, and ‘WeightScore’ in our analysis. The poor performance of the Thai model in our population demonstrates the importance of developing context relevant solutions to respond to the differences in epidemiology, ethics, and healthcare resources at a national level.

**FIGURE 4:**
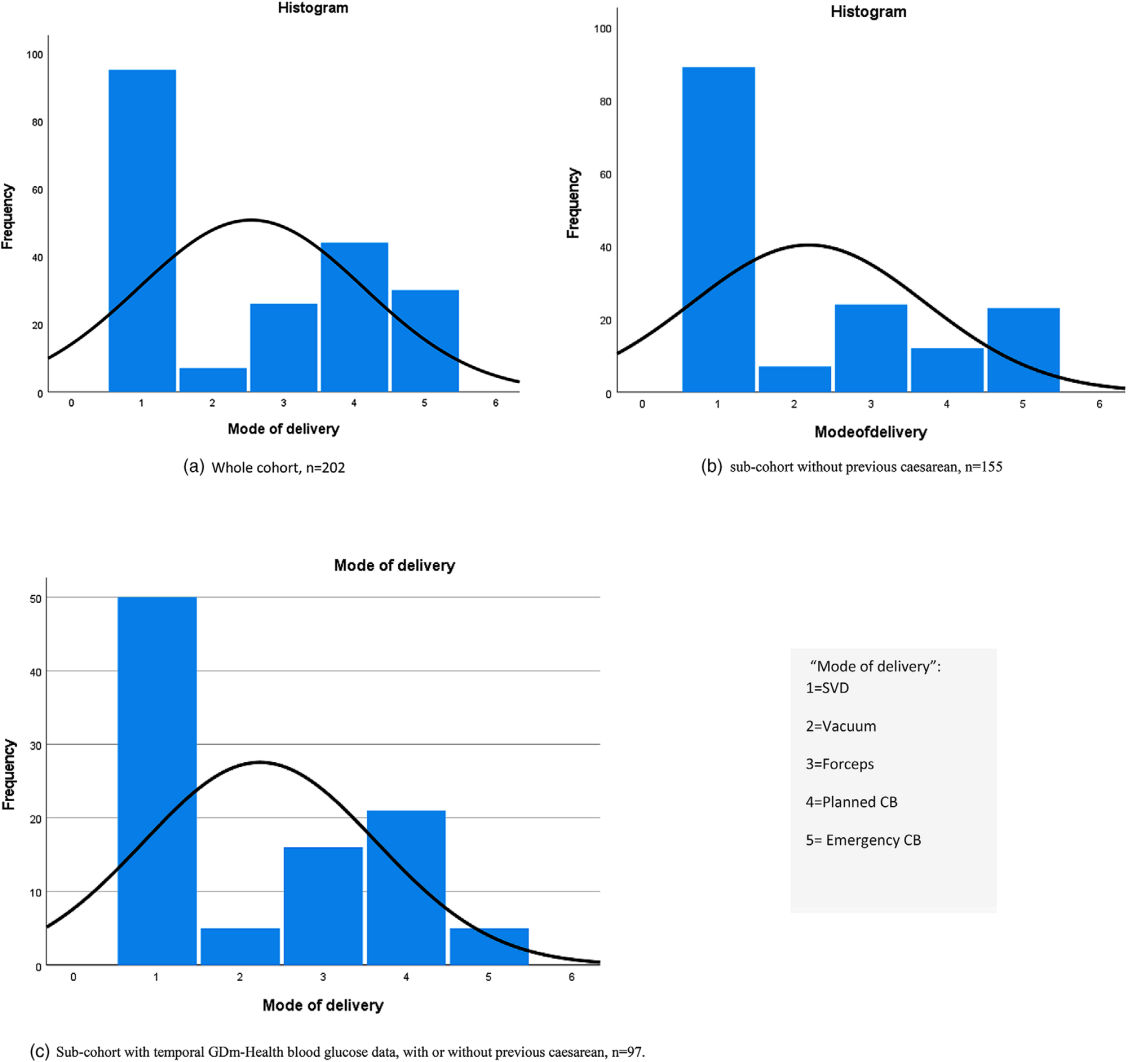
Distribution of delivery modes within the TREAT‐GDm cohort

The study also has limitations. Our data set was relatively small and we did not have an external validation set. Therefore, we cannot comment on the performance of our Oxford caesarean prediction score table in the other UK or international cohorts, nor on the overall false‐positive rate, sensitivity or specificity needed when considering new clinical prediction tools. Another limitation is that we did not develop a caesarean prediction table using temporal blood glucose (BG) measurements. Due to the data size, the machine learning models in model two only included BG measurements before breakfast. To include features for other BG time points, and for including engineered features, we need a larger dataset. Another limitation is that we have combined delivery modes SVD, vacuum, and forceps in the same delivery mode group to distinguish from caesarean birth. This grouping method introduces heterogeneity within the group. The distribution and the variation of delivery modes were observed in our study, as shown in Figure 4.

In future studies, we would like to carry out modelling analysis on a larger patient cohort to confirm the association between blood glucose profiles during pregnancy and the likelihood of caesarean birth, and develop a caesarean prediction score for both mothers with or without previous caesarean section. A comprehensive table can assist clinic decision on caesarean planning, and advise the roles of diabetes and its management in blood glucose and medication, standardise the assessment of caesarean birth, reduce the amount of ECB, and thereby improve the quality of care for mothers with GDM.

## CONFLICT OF INTEREST

L.M. is supported by the NIHR Oxford Biomedical Research Centre and is a part‐time employee of Sensyne Health plc.

## FUNDING INFORMATION

This work was funded by the Royal Academy of Engineering (KHDMM280122‐001), Daphne Jackson Trust, Oxford John Fell Fund (0011028), Wellcome Trust (217650/Z/19/Z). The research was supported by the National Institute for Health Research (NIHR) Oxford Biomedical Research Centre (BRC). The views expressed are those of the authors and not necessarily those of the NHS, the NIHR or the Department of Health.

## Data Availability

Clinical Trail research data are not shared.
